# Differences in Spontaneously Avoiding or Approaching Mice Reflect Differences in CB1-Mediated Signaling of Dorsal Striatal Transmission

**DOI:** 10.1371/journal.pone.0033260

**Published:** 2012-03-08

**Authors:** Daniela Laricchiuta, Silvia Rossi, Alessandra Musella, Valentina De Chiara, Debora Cutuli, Diego Centonze, Laura Petrosini

**Affiliations:** 1 Centro Europeo per la Ricerca sul Cervello (CERC)/Fondazione Santa Lucia, Rome, Italy; 2 Dipartimento di Psicologia, Università “Sapienza” di Roma, Rome, Italy; 3 Clinica Neurologica, Dipartimento di Neuroscienze, Università Tor Vergata, Rome, Italy; Catholic University of Sacred Heart of Rome, Italy

## Abstract

Approach or avoidance behaviors are accompanied by perceptual vigilance for, affective reactivity to and behavioral predisposition towards rewarding or punitive stimuli, respectively. We detected three subpopulations of C57BL/6J mice that responded with avoiding, balancing or approaching behaviors not induced by any experimental manipulation but spontaneously displayed in an approach/avoidance conflict task. Although the detailed neuronal mechanisms underlying the balancing between approach and avoidance are not fully clarified, there is growing evidence that endocannabinoid system (ECS) plays a critical role in the control of these balancing actions. The sensitivity of dorsal striatal synapses to the activation of cannabinoid CB1 receptors was investigated in the subpopulations of spontaneously avoiding, balancing or approaching mice. Avoiding animals displayed decreased control of CB1 receptors on GABAergic striatal transmission and in parallel increase of behavioral inhibition. Conversely, approaching animals exhibited increased control of CB1 receptors and in parallel increase of explorative behavior. Balancing animals reacted with balanced responses between approach and avoidance patterns. Treating avoiding animals with URB597 (fatty acid amide hydrolase inhibitor) or approaching animals with AM251 (CB1 receptor inverse agonist) reverted their respective behavioral and electrophysiological patterns. Therefore, enhanced or reduced CB1-mediated control on dorsal striatal transmission represents the synaptic hallmark of the approach or avoidance behavior, respectively. Thus, the opposite spontaneous responses to conflicting stimuli are modulated by a different involvement of endocannabinoid signaling of dorsal striatal neurons in the range of temperamental traits related to individual differences.

## Introduction

The super-ordinate division of emotions is distributed along a bipolar dimension of affective valence, from approaching to avoiding stimuli [1], [2]. In particular, approach and avoidance motivation are defined as the energization of behavior by, or the direction of behavior towards or away from, positive or negative stimuli (objects, events, possibilities), respectively [1], [2]. Approach and avoidance temperaments are both accompanied by neurobiological sensitivity to, perceptual vigilance for, affective reactivity to stimuli, so that a stimulus positively or negatively evaluated produces motivation and effort to approach or avoid it. Given approach/avoidance discrimination is the primary and most elemental reaction to environmental inputs, all organisms produce constitutionally ingrained approach-avoidance responses [1], [3]. Approaching or avoiding new situations, objects or foods as well as counterbalancing each other to maintain reactions to unfamiliar stimuli within adaptive boundaries are integral to successful adaptation [4], [5]. Excessive approaching or avoiding behavior can lead to psychopathological disorders, including depression, anxiety and addiction [6]–[8].

There is growing evidence that endocannabinoid system (ECS) plays an important role in the balancing control between approach and avoidance both in humans [9], [10] and rodents [5], [11], but its detailed action mechanism is not fully clarified. ECS is involved in tuning behaviors mediated by the reward central networks [12]–[14] and in particular in the rewarding properties of palatable foods [15], [16]. ECS is formed by cannabinoid receptors, their endogenous lipid ligands (endocannabinoids) and the machinery for synthesis and degradation of endocannabinoids [17]. Most central ECS functions are mediated by cannabinoid type-1 receptors (CB1) [14], [17], [18], densely expressed in numerous brain regions, as neocortex, basal ganglia, amygdala, hippocampus, hypothalamus and cerebellum [19]–[21]. CB1 receptors presynaptically inhibit both glutamatergic and GABAergic neurotransmission [12], [22], [23]. Such an inhibitory control on different neuronal subtypes would determine the bimodal effects of endocannabinoids on food intake, effects dependent also on their concentration [24].

In rodents, cocaine-induced conditioned place preference as well as running wheel spontaneous activity or sucrose consumption (manipulations with strong rewarding and reinforcing properties) are associated with hypersensitivity of striatal GABAergic synapses to CB1 receptors stimulation [25], [26]. These findings raise the intriguing possibility that even spontaneous forms of reward-based behaviors may rely on the sensitization of CB1 receptor-mediated GABAergic transmission in the striatum. The present research was aimed at studying approach/avoidance behaviors related to seeking for a novel palatable food and their electrophysiological neuronal substrates. Given we were searching for individual differences in a spontaneous behavior not induced by any experimental manipulation, the behavior of adolescent mice in a conflict task able to reveal temperamental traits of approach or avoidance was analyzed. Adolescent subjects have been retained the most appropriate sample because they are reported to be statistically over-represented, when compared to adults, in the group showing prominent vulnerability to conflicting situations [27]–[31]. Notably, the individual behavioral differences emergent in adolescence are persistent traits maintained across the life-span, although modulated by environmental experiences [32], [33].

In the present study, the spontaneous behavior of mice submitted to an approach/avoidance conflict task and the CB1-mediated transmission in spiny neurons of the dorso-medial striatum, structure crucially involved in motivated and goal-directed behaviors [34]–[37], were analyzed.

## Results

### A/A Y-Maze

**Figure 1:**
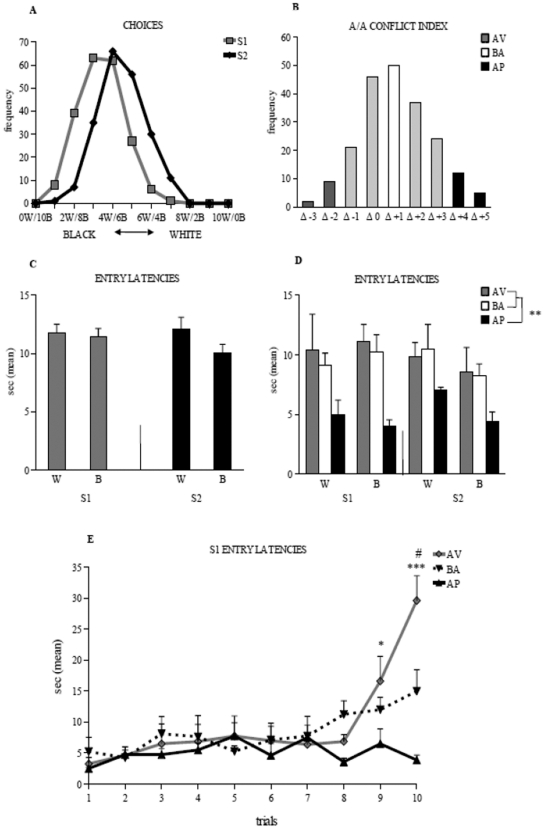
Responses to conflicting stimuli of adolescent mice in A/A Y-Maze. (**A**) Curves of distribution of the white and black choices of 206 animals during the sessions. (**B**) Curve of distribution of the A/A conflict index, considered as the difference (Δ) in the number of white choices between sessions. Entry latencies in the white and black arms of the entire sample of animals (**C**) and of the avoiding, balancing and approaching animals (**D**) during sessions, as well as latencies in the ten trials of S1 (**E**) in the three categories of animals are depicted. Abbreviations: W: white arm; B: black arm; S1: first session; S2: second session; AV: avoiding animals; BA: balancing animals; AP: approaching animals. In **C**, **D**, **E** and in the following figures, data are presented as means ± SEM. Asterisks indicate the significance level of the *post hoc* comparisons: in (**D**) AP *vs.* AV or BA groups: ** *P*<0.01; in (**E**) AV *vs.* AP groups: * *P*<0.05, *** *P*<0.0005; AV *vs.* BA groups: # *P*<0.05.

In both S1 and S2, the choices of 206 animals fitted the normal distribution fairly well. When both arms were rewarded with the same standard food (S1), most entries the animals made were in the reassuring black arm (1360 black choices versus 700 white choices out of 2060 total entries). Interestingly, when the aversive white arm was rewarded with the palatable food (S2), the distribution curve shifted towards the white choices (921 white choices out of 2060 total entries) (Fig. 1A). The frequencies of white choices in S1 and S2 showed a highly significant difference between sessions (χ^2^ = 272.23, *P*<0.00001).

Even the A/A conflict index was normally distributed (Fig. 1B) and its bell-shaped curve indicated that in S2 the palatable food, even if placed in the aversive white environment, was salient enough to increase white choices number (mean = Δ+1, *SD* = ±1.67). Thus, in the presence of conflicting inputs, we identified animals belonging to three behavioral categories, avoiding (AV), balancing (BA) and approaching (AP) animals. BA animals (24% of the sample) reacted with balancing responses between approach and avoidance and their values corresponded to the mean. The two opposite curve tails represented the few subjects exhibiting responses unbalanced towards one of the conflicting inputs: AV animals (5%) whose values were minus two *SD* of the mean, exhibited avoidance responses to the conflicting stimuli, while AP animals (8%) whose values were plus two *SD* of the mean, displayed approach responses to the conflicting stimuli. Differences in body weight did not influence the behavioral category animals belonged to (Two-way ANOVA on body weight: category: *F*_2,21_ = 0.01, *P* = 0.98; session: *F*_1,21_ = 2.81, *P* = 0.11; interaction: *F*_2,21_ = 1.92, *P* = 0.17), strongly indicating that the runts of the litter were not the more likely avoiding mice and “king-size” pups were not the more likely approaching animals.

A two-way ANOVA (arm×session) on the entry latencies of the 206 animals failed to indicate significant arm (*F*_1,205_ = 0.54, *P* = 0.46) and session (*F*_1,205_ = 3.39, *P* = 0.07) effects. Also interaction was not significant (*F*_1,205_ = 1.75, *P* = 0.19) (Fig. 1C). When entry latencies were analyzed in relation to the categories the animals belonged to, faceted results were found (Fig. 1D). A three-way ANOVA (category×arm×session) revealed a significant category effect (*F*_2, 21_ = 9.18, *P* = 0.001), while arm (*F*_1,21_ = 0.07, *P* = 0.799) and session (*F*_1, 21_ = 1.5, *P* = 0.23) effects were not significant. First- and second-order interactions were not significant. *Post hoc* comparisons on category effect revealed significant differences between AP and BA (*P* = 0.005) or AV (*P* = 0.002) animals and no difference between AV and BA animals (*P* = 0.9).

By analyzing the entry latencies regardless arm color or reward in the ten trials of the two sessions, in S1 the three categories of animals started with very similar values, increased throughout the session in AV and BA groups, but not in AP group and reached significant differences in AV animals in the last two trials (Fig. 1E) (Two-way ANOVA: category: *F*_2,21_ = 5.73, *P* = 0.01; trial: *F*_9,189_ = 5.5, *P*<0.00001; interaction: *F*_18,189_ = 2.42, *P* = 0.001). No differences in latency values were found in S2 (Two-way ANOVA: category: *F*_2,21_ = 1.85, *P* = 0.2; trial: *F*_9,189_ = 2.93, *P* = 0.003; interaction: *F*_18,189_ = 0.7, *P* = 0.8).

### OF

**Figure 2:**
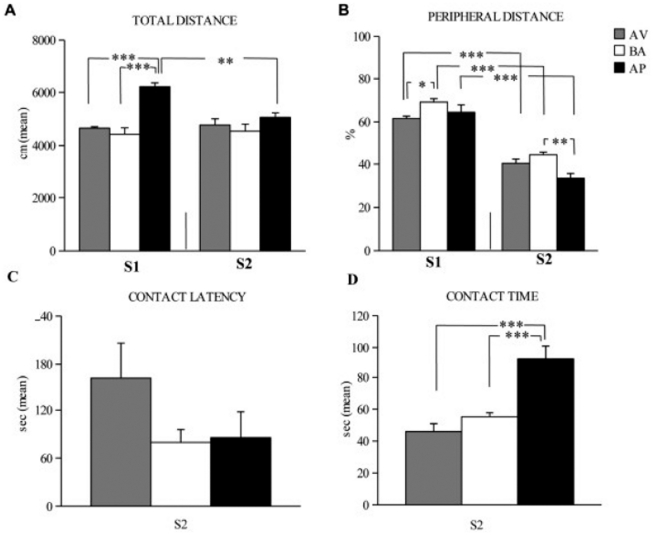
Responses of avoiding (AV), balancing (BA), and approaching (AP) mice in OF test. In S1, AP animals exhibited longer total distances (**A**) than AV and BA animals. In S2, AP mice spent more time in contacting the object (**D**), reduced total (**A**) and peripheral (**B**) distances. AV and BA animals displayed similar response patterns in almost all OF parameters (**A–D**). Abbreviations: S1: first session; S2: second session. Asterisks indicate the significance level of the *post hoc* comparisons between groups: * *P*<0.05; ** *P*<0.005; *** *P*<0.0005.

In the OF, AP animals were more active, rapid and explorative in moving into the environment than BA and AV animals, as revealed by two-way ANOVAs (category×session) on OF explorative parameters (total distance: category: *F*_2,21_ = 15.47, *P* = 0.0001; session: *F*_1,21_ = 4.84, *P* = 0.04; interaction: *F*_2,21_ = 9.25, *P* = 0.0013; peripheral distance: category (*F*_2,21_ = 4.97, *P* = 0.02; session (*F*_1,21_ = 398.76, *P*<0.00001; interaction (*F*_2,21_ = 4.90, *P* = 0.02). Furthermore, the AP animals were more active than BA and AV animals in contacting the object, as revealed by one-way ANOVA on contact time (*F*_2,21_ = 19.99, *P* = 0.00001). One-way ANOVA on contact latency did not reveal significant differences among animals (*F*_2,21_ = 1.77, *P* = 0.2). *Post hoc* comparisons are reported in Fig. 2A–D.

**Figure 3:**
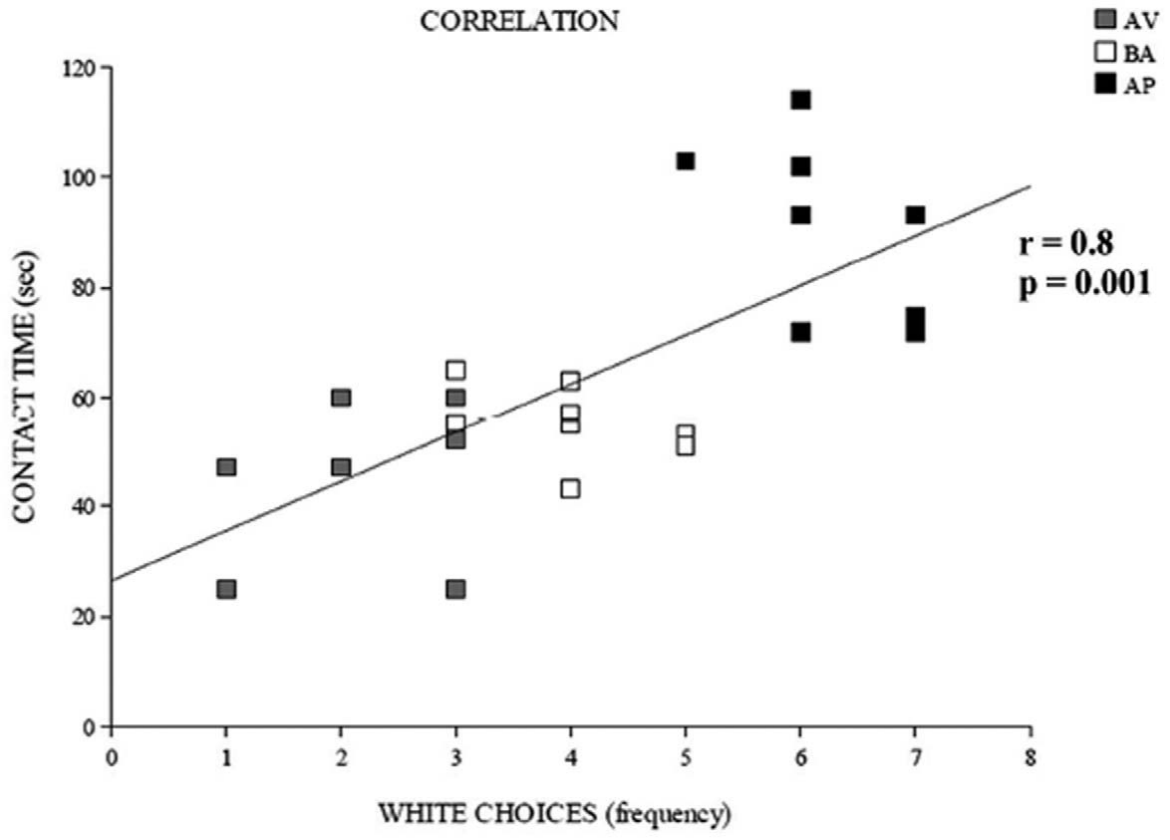
Correlation between frequency of white choices in the A/A Y-Maze (abscissa) and contact time of object in the OF (ordinate). A positive significant correlation has showed in the scatter plot. Abbreviations: AV: avoiding animals; BA: balancing animals; AP: approaching animals.

A positive significant correlation between frequency of white choices in the A/A Y-Maze and contact time of object in the OF was found (*r* = 0.8, *P* = 0.001) (Fig. 3).

### Electrophysiological recordings

**Figure 4:**
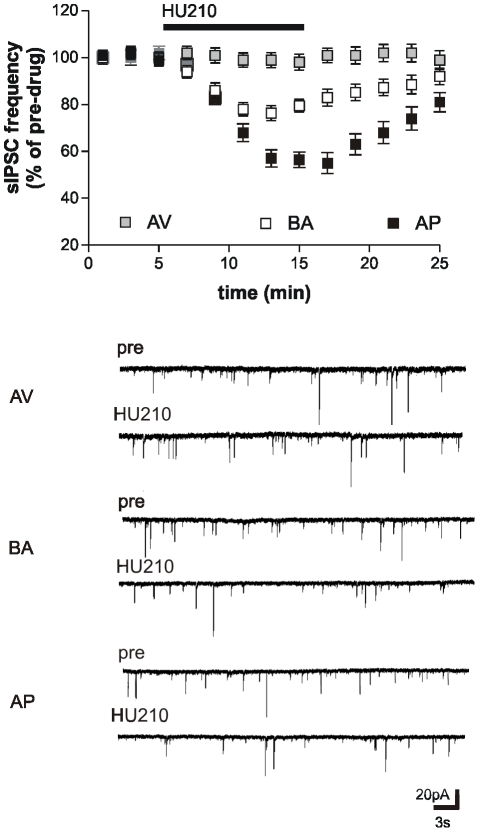
Responses of striatal neurons of avoiding (AV), balancing (BA), and approaching (AP) animals. The graph shows that the sIPSC frequency reduction induced by cannabinoid CB1 receptor agonist HU210 in the BA was potentiated in the AP and abolished in neurons from the AV animals. The electrophysiological traces on the bottom are examples of voltage-clamp recordings before and during the application of HU210 in AV, BA, and AP animals.

Striatal neurons recorded from AV, BA and AP animals displayed remarkably different responses to the stimulation of CB1 receptors (Fig. 4). In fact, bath application of the CB1 receptor agonist HU210 (10 min) caused a significant inhibition of sIPSCs frequency only in neurons from the BA animals (paired Student's *t*-test: *P*<0.05, n = 18, compared with pre-HU210 values) and AP animals (paired Student's *t*-test: *P*<0.05, n = 11, compared with pre-HU210 values), while it did not cause any effect in neurons from the AV group (paired Student's *t*-test: *P*>0.05, n = 12, compared with pre-HU210 values). The effect of HU210 in the three categories of animals was analyzed by one-way ANOVA (*F*_2,38_ = 21.5, *P*<0.0001). *Post hoc* comparisons showed that HU210 responses of AV animals were significantly different from that showed by AP (*P*<0.01) and BA animals (*P*<0.01). Notably, HU210 responses were enhanced in AP when compared to BA animals (*P*<0.01).

Furthermore, blockade of CB1 receptors with AM251 bath application (10 min) failed to enhance sIPSC frequency in neurons from the three experimental groups (paired Student's *t*-test: *P*>0.05, n = 6, compared to pre-AM251 values for AV, BA, and AP mice), ruling out that a different endocannabinoid tone may account for their different sensitivity to HU210 in these mice.

**Figure 5:**
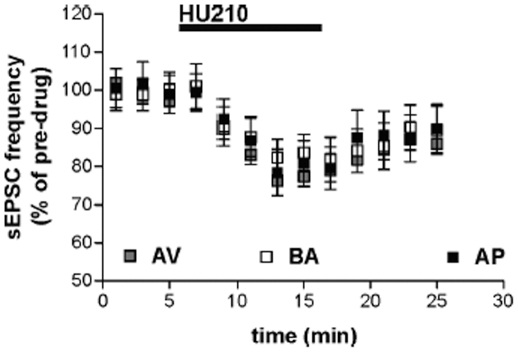
Responses of striatal neurons of avoiding (AV), balancing (BA), and approaching (AP) animals. The graph shows that the sEPSC frequency reduction induced by cannabinoid CB1 receptor agonist HU210 was analogous in the AV, BA, and AP animals.

CB1 receptors also control glutamate transmission in the striatum by a presynaptic mechanism [25], [26], [38]. HU210 inhibited glutamate-mediated sEPSC frequency to a similar extent (Fig. 5) (paired Student's *t*-test: *P*>0.05 compared with pre-HU210 values) in slices from AV (n = 5), BA (n = 9), and AP (n = 6) animals, indicating that only the sensitivity of CB1 receptors regulating GABA synapses is different in the three experimental groups.

These results, therefore, indicate that enhanced or reduced CB1 receptor control on GABAergic transmission represents the synaptic hallmarks of the approach or avoidance behavior, respectively.

### Behavioral effects of drugs acting on ECS

**Figure 6:**
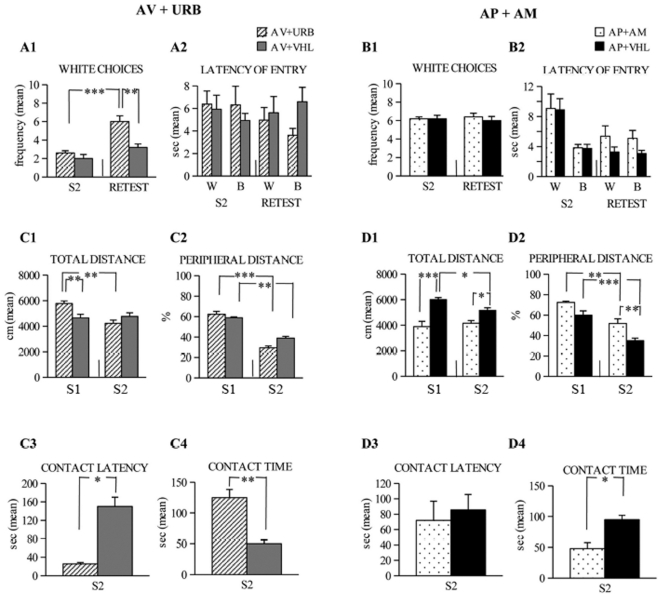
Behavioral responses of avoiding (AV) and approaching (AP) mice in A/A Y-Maze and OF. On the left side, the responses of AV animals treated with URB597 (URB) or vehicle (VHL) in A/A Y-Maze (**A1**, **A2**) and OF (**C1–C4**). AV+URB animals displayed enhancement of white choices and decrease of entry latencies mainly in entering the black arm, while they were more active in exploring the environment and contacting the object in the OF than AV+VHL animals. On the right side, the responses of AP animals treated with AM251 (AM) or vehicle (VHL) in A/A Y-Maze (**B1**, **B2**) and OF (**D1–D4**). AP+AM did not change the number of white choices, decreased entry latencies in the white arm in A/A Y-Maze, while they were less active in exploring the environment and contacting the object in the OF than AP+VHL animals. Abbreviations: W: white arm; B: black arm; S1: first session; S2: second session. Asterisks indicate the significance level of the *post hoc* comparisons between groups: * *P*<0.05; ** *P*<0.005; *** *P*<0.0005.

To further analyze ECS functioning in the animals belonging to the extreme AV and AP categories, we tested them under the action of URB597 or AM251 respectively, in the A/A Y-maze and OF tasks. The results obtained from AV animals treated with URB597 or VHL in the re-test session of A/A Y-maze were compared with those they had displayed in the previous S2. AV+URB animals significantly increased the number of white choices, at odds with AV+VHL animals that maintained their avoiding behavior (Fig. 6 A1). Two-way ANOVA (drug×session) on white choices revealed significant drug (*F*_1,8_ = 8.89, *P* = 0.017) and session (*F*_1,8_ = 70.53, *P* = 0.00003) effects. Also the interaction was significant (*F*_1,8_ = 16.13, *P* = 0.003). Notably, this behavior was accompanied by decreased entry latencies, mainly in entering the black arm. A three-way ANOVA (drug×arm×session) on entry latencies revealed not significant main effects (drug: *F*_1,8_ = 0.16, *P* = 0.69; arm: *F*_1,8_ = 3.85, *P* = 0.08; session: *F*_1,8_ = 0.15, *P* = 0.70). The only significant interaction was the first-order interaction drug×arm (*F*_1,8_ = 15.12, *P* = 0.004) (Fig. 6 A2). Even in the OF, the AV+URB animals were more active than AV+VHL animals in exploring the environment and contacting the object (Two-way ANOVAs (drug×session): total distance: drug: *F*_1,8_ = 1.28, *P* = 0.29; session: *F*_1,8_ = 22.03, *P* = 0.0016; interaction: *F*_1,8_ = 29.8, *P* = 0.0006; peripheral distance: drug: *F*_1,8_ = 7.24, *P* = 0.02; session: *F*_1,8_ = 121.04, *P*<0.00001; interaction: *F*_1,8_ = 5.49, *P* = 0.04; One-way ANOVAs: contact latency: drug: *F*_1,8_ = 6.07, *P* = 0.039; contact time: drug: *F*_1,8_ = 29.83, *P* = 0.0006). *Post hoc* comparisons are reported in Fig. 6, C1–C4.

Accordingly, the data of AP animals tested under the action of AM251 or VHL in the re-test session of A/A Y-maze were compared with those they had displayed in the previous S2. AP+AM animals did not change their number of white choices (Fig. 6 B1) likely the AP+VHL animals, as revealed by a two-way ANOVA (drug: *F*_1,8_ = 0.2, *P* = 0.66; session: *F*_1,8_ = 0.00, *P* = 1; interaction: *F*_1,8_ = 0.57, *P* = 0.47). This behavior was accompanied by a significant decrease of entry latencies in the white arm (Three-way ANOVA: drug: *F*_1,8_ = 0.80, *P* = 0.39; arm: *F*_1,8_ = 14.70, *P* = 0.004; session: *F*_1,8_ = 20.49, *P* = 0.001; the only significant interaction was arm×session *F*_1,8_ = 21.53, *P* = 0.001) (Fig. 6 B2). In the OF, AP+AM animals were less active than AP+VHL animals in exploring the environment and contacting the object (Two-way ANOVAs (drug×session): total distance: drug: *F*_1,8_ = 24.54, *P* = 0.001; session: *F*_1,8_ = 6.48, *P* = 0.034; interaction: *F*_1,8_ = 16.66, *P* = 0.003; peripheral distance: drug: *F*_1,8_ = 9.70, *P* = 0.01; session: *F*_1,8_ = 146.35, *P*<0.00001; interaction: *F*_1,8_ = 5.92, *P* = 0.04; One-way ANOVAs: contact latency: drug: *F*_1,8_ = 0.09, *P* = 0.77; contact time: drug: *F*_1,8_ = 11.07, *P* = 0.01). *Post hoc* comparisons are reported in Fig. 6, D1–D4.

As further verification of these effects, we counterbalanced the pharmacological manipulations between categories, by analyzing URB597 effects in five different AP animals as well as AM251 effects in five different AV animals, in A/A Y-maze and OF tasks.

**Figure 7:**
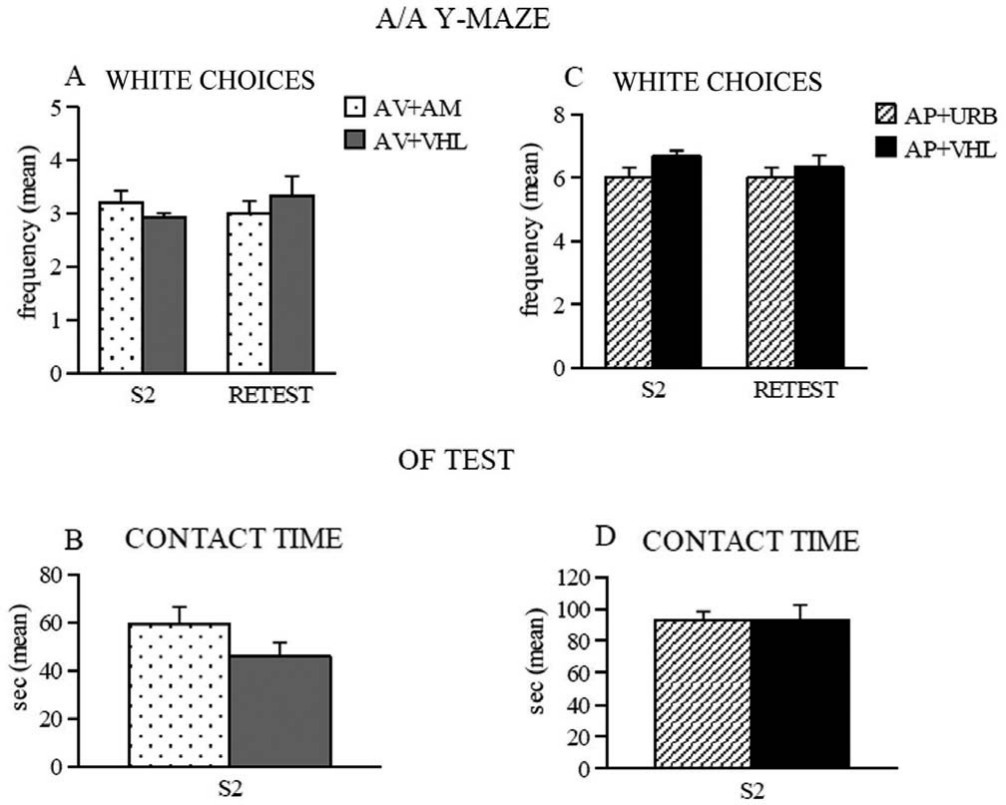
Behavioral responses of avoiding (AV) and approaching (AP) mice in A/A Y-Maze and OF. On the left side, the responses of AV animals treated with AM251 (AM) or vehicle (VHL) in A/A Y-Maze (**A**) and OF test (**B**). In A/A Y-Maze, AV+AM animals maintained the number of white choices and in the OF test they explored the environment and contacted the object not differently than AV+VHL animals. On the right side, the responses of AP animals treated with URB597 (URB) or vehicle (VHL) in A/A Y-Maze (**C**) and OF test (**D**). In A/A Y-Maze, AP+URB animals did not change the number of white choices and in the OF test they were active as AP+VHL animals in exploring the environment and contacting the object. Abbreviations: S2: second session; retest: retest session.

The results obtained from AV animals treated with AM251 or VHL in the re-test session of A/A Y-maze were compared with those they had displayed in the previous S2. AV+AM animals maintained their number of white choices, as AV+VHL animals did (Fig. 7A). This behavior was accompanied by unmodified entry latencies, in entering both arms (Two-way ANOVA (drug×session) on white choices: drug: *F*_1,8_ = 0.02, *P* = 0.88; session: *F*_1,8_ = 0.20, *P* = 0.66; interaction: *F*_1,8_ = 1.89, *P* = 0.20; Three-way ANOVA (drug×arm×session) on entry latencies: drug: *F*_1,8_ = 0.12, *P* = 0.73; arm: *F*_1,8_ = 1.39, *P* = 0.27; session: *F*_1,8_ = 0.35, *P* = 0.56; first- or second-order interactions were all not significant).

In the OF test, AV+AM animals explored the environment not differently from AV+VHL animals and did not modify the contact with the new object (Fig. 7B) (ANOVAs (drug×session): total distance: drug: *F*_1,8_ = 0.71, *P* = 0.42; session: *F*_1,8_ = 2.05, *P* = 0.19; interaction: *F*_1,8_ = 0.95, *P* = 0.36; peripheral distance: drug: *F*_1,8_ = 1.17, *P* = 0.31; session: *F*_1,8_ = 210.79, *P* = 0.00001; interaction: *F*_1,8_ = 0.004, *P* = 0.95; One-way ANOVAs: contact latency: drug: *F*_1,8_ = 5.05, *P* = 0.06; Contact time: drug: *F*_1,8_ = 2.28, *P* = 0.16).

Analogously, the data of AP animals tested under the action of URB597 or VHL in the re-test session of A/A Y-maze were compared with those they had displayed in the previous S2. As AP+VHL animals, AP+URB animals did not significantly change their number of white choices (Two-way ANOVA: drug: *F*_1,8_ = 1.66, *P* = 0.23; session: *F*_1,8_ = 0.84, *P* = 0.38; interaction: *F*_1,8_ = 0.84, *P* = 0.38) (Fig. 7C) and did not modify entry latencies in entering both arms (Three-way ANOVA: drug: *F*_1,8_ = 1.01, *P* = 0.34; arm: *F*_1,8_ = 1.77, *P* = 0.21; session: *F*_1,8_ = 0.003, *P* = 0.95; first- or second-order interactions were not significant).

In the OF test, AP+URB animals were active as AP+VHL animals in exploring the environment and contacting the new object (Fig. 7D) (ANOVAs (drug×session): total distance: drug: *F*_1,8_ = 0.15, *P* = 0.70; session: *F*_1,8_ = 36.03, *P* = 0.0003; interaction: *F*_1,8_ = 3.11, *P* = 0.11; peripheral distance: drug: *F*_1,8_ = 0.23, *P* = 0.64; session: *F*_1,8_ = 200.20, *P* = 0.00001; interaction: *F*_1,8_ = 2.08, *P* = 0.18; One-way ANOVAs: contact latency: drug: *F*_1,8_ = 2.49, *P* = 0.15; contact time: drug: *F*_1,8_ = 0.0009, *P* = 0.97).

### Electrophysiological effects of drugs acting on ECS

In line with the behavioral data and with previous findings [39], the treatment of AV animals with URB597 was able to rescue the sensitivity of striatal GABAergic synapses to HU210 (paired Student's *t*-test: *P*<0.05, n = 6 compared with pre-HU210 values). On the other hand, blockade of CB1 receptors with AM251 fully abolished HU210 responses in AP mice (paired Student's *t*-test: *P*>0.05, n = 8 compared with pre-HU210 values).

**Figure 8:**
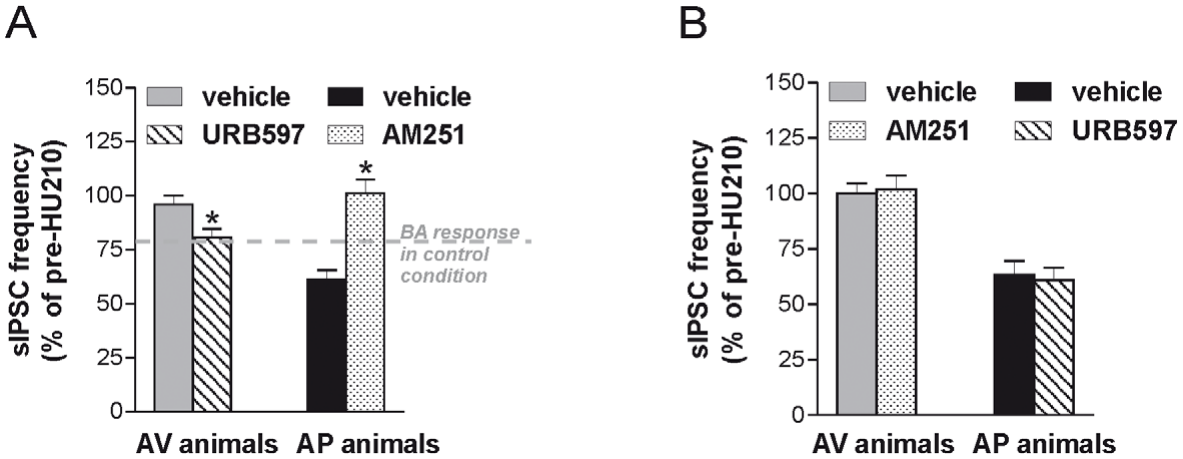
Electrophysiological effects of drugs acting on endocannabinoid system in avoiding (AV) and approaching (AP) mice. (**A**) The graph shows that treatment with URB597 was able to rescue the effect of the CB1 receptor agonist HU210 on sIPSC frequency in neurons from the AV animals. The HU210-induced inhibition of sIPSC frequency in AV+URB animals was comparable to that seen in BA animals. The treatment with the CB1 receptor antagonist AM251 fully abolished HU210 responses in AP mice. (**B**) The counterbalanced pharmacological manipulations between categories showed that in AV mice blockade of CB1 receptors with AM251 did not modify HU210 responses of GABAergic striatal neurons, while the treatment of AP animals with URB maintained the high sensitivity of striatal GABAergic synapses to HU210. Asterisks indicate the significance level of the comparisons between AV+URB *vs.* AV+VHL animals and AP+AM *vs.* AP+VHL animals: * *P*<0.05.

As a further verification of the described effects, we counterbalanced the pharmacological manipulations between categories, by analyzing the effects of URB597 in five different AP animals as well as the effect of AM251 in five different AV animals. Blockade of CB1 receptors with AM251 did not modify HU210 responses of GABAergic striatal neurons in AV mice (paired Student's *t*-test: *P*>0.05, n = 5 compared with pre-HU210 values), while the treatment of AP animals with URB597 maintained the high sensitivity of striatal GABAergic synapses to HU210 (paired Student's *t*-test: *P*<0.05, n = 6 compared with pre-HU210 values) (Fig. 8).

### Anxiety level evaluation

**Figure 9:**
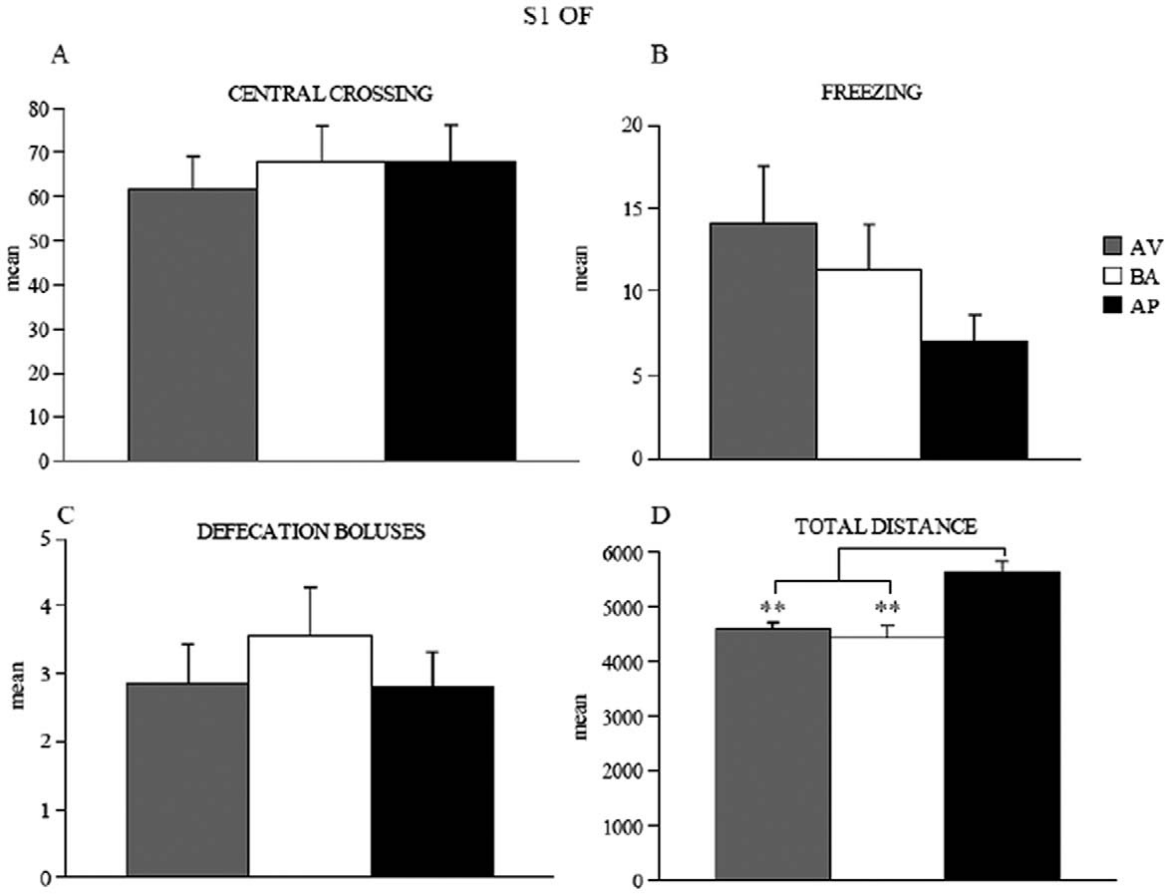
Behavioral responses of avoiding (AV), balancing (BA), and approaching (AP) mice in the S1 of OF. Central crossings (**A**), freezing (**B**), defecation boluses (**C**), total distance (**D**) are depicted. Asterisk indicates the significance level of the *post hoc* comparisons between groups: ** *P*<0.01.

To address whether anxiety might contribute to avoidance and approach patterns, the behaviors exhibited by AP, BA and AV animals (8/category) were evaluated considering parameters more directly linked to anxiety. The OF parameters linked to anxiety were measured in S1 and revealed no difference among categories (Fig. 9A–D) (One-way ANOVAs: central crossings: *F*_2,21_ = 0.18, *P* = 0.83; freezing: *F*_2,21_ = 1.97, *P* = 0.16; defecation boluses: *F*_2,21_ = 0.5, *P* = 0.61). Anyway, the already noted differences among AV, BA and AP animals in parameters related to exploration were again found. In fact, AP animals explored the environment significantly more than the remaining AV and BA animals (One-way ANOVA on total distance: *F*_2,21_ = 12.86, *P* = 0.0002).

**Figure 10:**
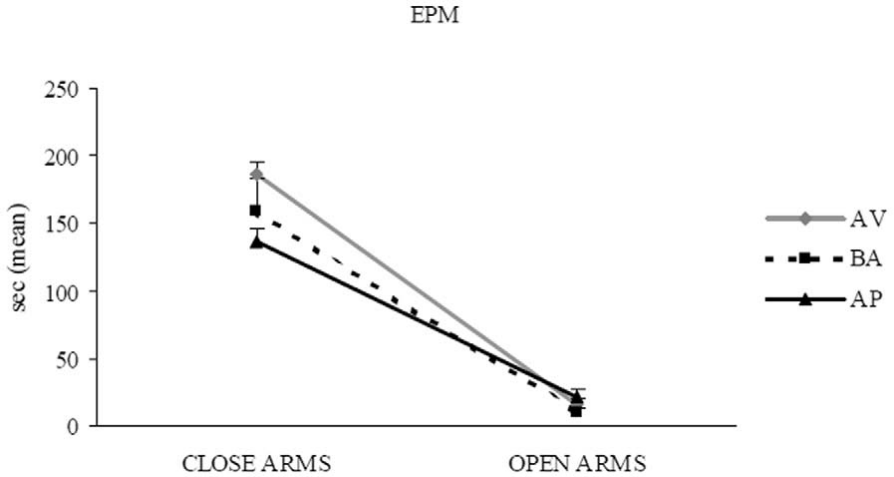
Behavioral responses of avoiding (AV), balancing (BA), and approaching (AP) mice in the EPM. All animals regardless the category they belonged to spent more time in the close arms, exhibiting thus the normal open arm avoidance.

In the EPM (Fig. 10), all animals regardless the category they belonged to spent more time in the close arms, exhibiting thus the normal open arm avoidance (Two-way ANOVA: category: *F*_2,21_ = 0.73, *P* = 0.48; arm: *F*_1,21_ = 449.22, *P*<0.00001; interaction: *F*_2,21_ = 2.35, *P* = 0.11). Furthermore, no significant difference in defecation boluses was found among groups (ANOVA: category: *F*_2,21_ = 0.28, *P* = 0.75; arm: *F*_1,21_ = 33.71, *P*<0.00001; interaction: *F*_2,21_ = 0.28, *P* = 0.75).

## Discussion

The present study demonstrates a different control of striatal CB1 receptors on GABAergic neurotransmission in relation to individual differences in the spontaneous response of approach or avoidance to conflicting stimuli and advances a framework for explaining behaviors of approach and avoidance involving the ECS at striatal level.

Approach system is a motivational system activating reward-seeking behavior associated with impulsivity/exploration, while avoidance system is an attentional system promoting inhibition of appetitive responses [40], [41]. Excessive reactivity of these systems has been related to psychopathological disorders, as attention-deficit/hyperactivity disorders (ADHD) or depression on one hand and anxiety on the other hand [7], [8], [42], [43]. The innovative use of experimental protocols made in the current research allowed analyzing spontaneous motivational components that guide behavioral responses ranging from approaching to avoiding stimuli. In the presence of the same conflicting stimuli, while BA animals reacted with balanced responses between approach and avoidance, AV or AP animals respectively exhibited inhibitory or approach responses towards one of the conflicting inputs.

The behavioral differences observed in AV or AP animals were not linked to different levels of anxiety. Conversely, the behaviors linked to exploration and approach were significantly influenced by the category the mice belonged to. Namely, AP mice in A/A Y-Maze displayed the lowest entry latencies and in the OF traveled the longest distances in the arena, contacted longer the object, demonstrating they were indeed approaching and explorative. Furthermore, in S1 ten trials of A/A Y-Maze test, all animals exhibited similar anxiety levels in the very first trials (the latencies of all animals started with very similar values), AP animals were the only subjects maintaining high reactivity when the trials went by (entry latencies progressively increased only in AV and BA groups), at the end of the task AV animals made evident a behavioral inhibition (in the last two trials their latencies reached values significantly higher).

The relation between reward-seeking behavioral activation and exploration/impulsivity has been also found in previous studies reporting that impulsivity and extraversion [44], [45], as well as risk aversion and low motivation [46], [47] are related to each other.

Intriguingly, the individual differences in temperamental traits were reflected in the differences in the CB1-mediated activity of the dorsal striatal network. In fact, CB1-mediated presynaptic control on GABAergic transmission in the dorsal striatal medium spiny neurons was almost absent in AV animals and conversely increased in AP animals. ECS plays a central role in the balancing control between approach and avoidance in both humans [9], [10] and rodents [5], [11] and modulates GABAergic inhibition controlling fine-tuned behaviors [48]. Notably, reward-associated environmental manipulations sensitize CB1-mediated striatal transmission [25], [26], while chronic psycho-emotional stress causes marked down-regulation of CB1-controlled GABAergic striatal transmission [38]. Recent neuroimaging findings in healthy human subjects indicate striatal and prefrontal functional differences in reward processing related to differences in approach/avoidance personality traits [49], [50]. Individual differences on expectation and receipt of reward have been found also in clinical populations, demonstrating abnormal reward processing in psychopathological disorders, as bipolar mania [51], substance dependence [52], schizophrenia [51], [53], ADHD [54] and depression [55]. The present experimental findings fully fit with these functional studies.

In the AV animals the enhancement of the endogenous tone of anandamide (AEA) with URB597 increased number of white choices and decreased entry latencies in the A/A Y-maze as well as enhanced explorative behavior and contact times in the OF test. These behavioral responses were consistently paralleled by the rescue of CB1 receptor sensitivity to HU210, indicating that indeed striatal CB1 receptors modulate spontaneous reward-related processes. The intriguing observation that URB597 reinstates “sensitivity” to HU210 in AV mice deserves further discussion. AV mice fail to respond to HU210, suggesting silencing of CB1 receptors. How may the increase in endocannabinoid signaling by blocking AEA degradation reinstate CB1 control on GABAergic transmission? Notably, in the striatum the enhancement of AEA tone with URB597 inhibits sEPSC but not sIPSC frequency [39] because CB1 receptors controlling glutamate release are the target of AEA, while the other endocannabinoid 2-AG is the preferential endogenous agonist of CB1 receptors controlling GABAergic transmission. In fact, stimulation of 2-AG synthesis with DHPG [56], [57] or following acetylcholine M1 receptor activation [58] reduces GABAergic but not glutamatergic synaptic events. Thus, in AV animals the AEA increase, caused by URB597, reinstates the control of CB1 receptors on sIPSC frequency to HU210, indicating a complex interaction between the two main endocannabinoids and their receptors.

In AP animals the blockade of CB1 receptors with AM251 reduced contact times and explorative behavior in OF test, although it failed to affect white choice number in A/A Y-maze. Electrophysiological recordings in the same treated animals indicated a fully blocked CB1 receptor activity. Thus, AV or AP animals treated with ECS agonists or antagonists tended to fade away their behavioral features, rendering them less inhibited or less “triggered”, respectively. These findings are fully supported by counterbalancing the pharmacological manipulations in AV or AP animals. In fact, AV mice that had a reduced CB1 control on GABAergic neurotransmission when further inhibited by AM251 did not display any behavioral or electrophysiological modification. In parallel, AP animals that had an enhanced CB1 control on GABAergic neurotransmission when further potentiated by URB597 did not display any behavioral or electrophysiological modification.

ECS functional features and the pharmacological properties of the drugs acting on it provide a possible explanation for the different pharmacological efficacy found in the present research. In fact, endocannabinoids are synthesized and released “on demand” after neuronal depolarization [59], [60]. Indeed, by presynaptically reducing both excitatory and inhibitory neurotransmission, the ultimate effect of endocannabinoids depends on nature and amount of neurotransmitters being controlled [12], [14], [17], [22]–[24], [61]. The inhibition of endocannabinoid degradation by URB597 prolongs the neuronal signaling in active synapses only, preserving the spatio-temporal specificity of endocannabinoid activity [62]. Conversely, systemic CB1 receptor blockade by AM251 suppresses both excitatory and inhibitory ECS effects on multiple neuronal populations, explaining thus the different behavioral responses exhibited in the drug presence. Thus, the treatment with URB597 of the “behaviorally inhibited” AV animals enhanced the endocannabinoid tonic control over striatal GABAergic synapses and unhinged the behavioral inhibition featuring these animals. On the converse, the treatment with AM251 of the “explorative/impulsive” AP animals blocked the endocannabinoid tonic control over striatal synapses and prevented the triggered behavior featuring these animals. These findings are fully consistent with the decrease of anxious behaviors, the reduction of isolation-induced ultrasonic vocalizations in pups and the decrease of stress-induced corticosterone release provoked by URB597 injections [63]–[65]. Furthermore, they fit with the increase of the preference for palatable substances produced by administration of exogenous cannabinoids or endocannabinoids [66]–[68], and with the decrease in palatable food intake produced by treatment with AM251 [5], [23], [69].

The present research demonstrates that in responding to the same conflicting stimuli adolescent inbred mice exhibit variance of spontaneous behavior ranging from avoiding to approaching traits and that this behavioral variance is accompanied by a different CB1-mediated control on striatal neurotransmission. Human and rodent adolescents show a prominent motivation towards reward-responsivity, novelty seeking and impulsivity as well as increased vulnerability to affective illness and addiction [27], [29]–[31], [70]–[73]. Moreover, adolescent rats find repeated cannabinoid exposure less aversive than adult rats but exhibit memory deficits and changes in hippocampal protein expression more lasting [74]. Age-dependent differences in the brain levels of endocannabinoids as well as in CB1-mediated effects on synaptic transmission have been described [75]–[77]. However, the features linked to individual behavioral differences present in adolescence appear to be persistent traits maintained across the life-span [32], [33].

Interestingly, the individual predisposition to approach or avoidance demonstrated by the present data extends recent findings reporting differences in impulsivity associated with differences in striatum and nucleus accumbens monoamines [78] in inbred rodents. Phenotypic differences in susceptibility to stress associated with differences in responses to natural and drug rewards were also reported [32]. Because all same-sex members of inbred strains are genetically identical, when animals belonging to same strain are tested under controlled conditions, individual differences among animals have to reflect allelic and functional differences probably modulated by prenatal and postnatal environmental factors or early dominance hierarchies [79]–[81]. Although environmental influences determining the phenotypic variability in inbred subjects are difficult to control and measure, inbred mice raised in rigorously defined environments may show variability in some traits unrelated to genetic and environmental influences [82]. Future studies are needed to delineate the contribution of genetic, epigenetic and environmental variables that may together develop and modulate individual differences.

The behavioral responses to conflicting stimuli mirrored CB1-mediated control on dorsal striatal neuronal transmission. We are aware that the different CB1-mediated control on GABAergic transmission observed in the three behaviorally-characterized groups of animals could be linked to a different sensitivity of the CB1 receptor as an entity, or to differences in the distribution of CB1 receptors or to differences in the amount of CB1 receptors. Distinguishing among the three alternatives requires aimed studies with specific methodological approaches that are in progress. However, this drawback does not weaken the present data since the current findings do evidence that endocannabinoids acting on dorsal striatal neurons influence the spontaneous response to conflicting stimuli and support the involvement of endocannabinoid transmission in approach and avoidance behaviors that often feature not only full-blown psychiatric disorders but also the individual differences in non-pathological temperamental traits.

## Materials and Methods

### Subjects

Seven hundred eighty male adolescent (32±2 pnd) C57BL/6JOlaHsd mice (Harlan, Italy) were used. Description of subjects, experimental procedures and global timing of the experimental design are summarized in Fig. S1 and detailed in Materials and Methods S1.

### Ethics Statement

All efforts were made to minimize animal suffering and to reduce their number, in accordance with the European Community Council Directive of 24 November 1986 (86/609/EEC) and approved by the Ethical Committee on animal experiments of Santa Lucia Foundation.

### Behavioral Testing: Approach/Avoidance Y-Maze (A/A Y-Maze)

A Plexiglas Y-maze had a starting gray arm from which two arms (8×30×15 cm) stemmed, arranged at an angle of 90° to each other. A T-guillotine door was placed at the end of the starting arm to prevent the animal to be back. An arm entry was defined as four legs entering one of the arms. One of the two arms had black and opaque floor and walls and no light inside, while the other one had white floor and walls and was lighted by a 16-W neon lamp. The colored “furniture” as well as the neon lamp were exchangeable between arms to alternate the spatial position of the white and black arms. The apparatus was placed in a slightly lit room by a red light (40 W) and it was always cleaned thoroughly with 70% ethanol and dried after each trial to remove scent cues. At the end of each arm of choice there was a blue food tray (3 cm in diameter, 1 cm deep). The depth of the tray prevented mice from seeing the reward at a distance but allowed for an easy reward, i.e., eating as well as the appreciation of reward scent, not reducing the olfactory cues.

Since the appetites for palatable foods have to be learned [5], [83], a week before behavioral testing the animals were exposed to a novel palatable food (Fonzies, KP Snack Foods, Munchen, Germany) in their home cages for three consecutive days [84]. Fonzies (8% protein, 33% fat and 53% carbohydrate, for a caloric value of 541 kcal/100 gm) consisted of corn flour, hydrogenate vegetable fat, cheese powder and salt.

At the beginning of behavioral testing, mice were subjected to 1-day habituation phase in which all Y-Maze arms were opened to encourage maze exploration. During habituation, no food was present in the apparatus. To increase the motivation to search for the reward, 12 h before exposure to the experimental set-up, the animals were slightly food deprived by limiting the food access to 12 hours/day. Such a regimen resulted in no significant body weight loss. Mean values of weight ranged from 17.63±0.50 to 18.28±0.58 g. Testing phase consisted of two 10-trial sessions with 1 min-inter-trial interval. In the Session 1 (S1), the animal was placed in the

starting arm and could choose to enter one of the two arms, both containing the same standard food reward. At the end of each trial the reward was always replaced. The spatial position of each arm (black and dark or white and lighted) was side balanced during the whole test, to exclude any side preference.

During the Session 2 (S2) starting 24 h after S1, the white arm was rewarded with the highly palatable food (Fonzies), while the black arm was rewarded with the standard food pellet. Notably, this approach/avoidance test required to choose between two conflicting drives, reaching a palatable reward placed in an aversive (white and lighted) environment or reaching a standard food placed in a reassuring (black and dark) environment.

Parameters considered were: white choices, the frequency of entry into the white arm in S1 and S2; A/A conflict index, the difference (Δ) in the number of white choices between S1 and S2; entry latencies exhibited in white and black arms, separately or regardless arm color or reward in each trial of both S1 and S2.

### Behavioral Testing: Open Field Test With Novel Object (OF)

To eliminate the “food” and “palatability” dimensions and maintain the conflicting drives given by an appealing new object placed in an anxiogenic central location of a wide arena, OF test was used. The apparatus placed in a dimly lighted (red light 40 W) and soundproof cubicle room consisted of a circular arena (diameter 60 cm) delimited by a pale gray wall 20 cm high. In S1, a single animal was allowed to explore the empty open field and its baseline level of activity was measured. In S2, an object (a gray plastic cone: 10×6 cm; base diameter = 9.5 cm) was put in the arena center. Sessions lasted 10 min and inter-session interval was 5 min. The apparatus and the object were always cleaned thoroughly with 70% ethanol and dried after each session to remove scent cues. The whole testing was recorded by a video camera. The resulting video signal was relayed to a monitor and processed through an image analyzer (Ethovision, Noldus, Wageningen, The Netherlands).

The parameters considered were: total distance (in cm) traveled in the arena; peripheral distance, the percentage of total distance traveled in a 6-cm peripheral annulus; central crossings, number of entries into the 24-cm radius central area; duration of freezing, absence of all movements, (aside from those required for respiration); number of defecation boluses; contact latency with the object; contact time with the object. The contact with object was considered to take place when the mouse's snout actually touched the object, or when it sniffed the object for at least 1 sec.

### Behavioral Testing: Elevated Plus Maze (EPM)

EPM that exploits the natural aversion of rodents to heights and unprotected spaces consisted in a maze raised 90 cm above the ground formed by a wooden structure in the shape of a cross with four 30×5 cm arms extending from a central (5×5 cm) region. North and south arms were open, east and west arms were enclosed by 15 cm high walls. EPM behavioral indicators were: time spent in the open and closed arms; number of defecation boluses in the open and closed arms.

### Electrophysiological Recordings

Whole-cell patch clamp recordings from single striatal neurons in cortico-striatal coronal slices (200 µm) were performed according to previous studies [25], [26], [38], [85]. To detect spontaneous GABAA-mediated inhibitory postsynaptic currents (sIPSCs), intraelectrode solution had the following composition (mM): CsCl (110), K^+^-gluconate (30), ethylene glycol-bis (ß-aminoethyl ether)-N,N,N′,N′-tetra-acetic acid (EGTA; 1.1), HEPES (10), CaCl_2_ (0.1), Mg-ATP (4), Na-GTP (0.3). MK-801 (30 µM) and CNQX (10 µM) were added to the external solution to block, respectively, NMDA and nonNMDA glutamate receptors.

To study spontaneous glutamate-mediated excitatory postsynaptic currents (sEPSCs), the recording pipettes were filled with internal solution of the following composition: (mM) K^+^-gluconate (125), NaCl (10), CaCl_2_, (1.0), MgCl_2_ (2.0), 1,2-bis (2-aminophenoxy) ethane-N,N,N,N-tetraacetic acid (BAPTA; 0.5), N-(2-hydroxyethyl)-piperazine-N-s-ethanesulfonic acid (HEPES; 19), guanosine triphosphate (GTP; 0.3), Mg-adenosine triphosphate (Mg-ATP; 1.0), adjusted to pH 7.3 with KOH. Bicuculline (10 µM) was added to the perfusing solution to block GABAA-mediated transmission. sIPSCs or sEPSCs were stored by using P-CLAMP 9 (Molecular Devices, Foster City, CA, USA) and analyzed off line on a personal computer with Mini Analysis 5.1 (Synaptosoft, Leonia, NJ, USA) software. Offline analysis was carried out on sIPSCs or sEPSCs recorded during fixed times (5–10 samplings of 2–3 min duration each, recorded every 2–3 min). Only cells showing stable frequencies (<20% changes during the control samplings) were taken into account.

HU210 drug used in slices was first dissolved in DMSO, then in the bathing ACSF to the desired final concentration. DMSO alone was used in control experiments. The concentrations (µM) of the various drugs were as follows: AM251 (10), CNQX (10), HU210 (1), MK-801 (30) (Tocris, Bristol, UK), Bicuculline (10) (Sigma-RBI, St Louis, MO, USA).

For electrophysiological data, ‘n’ refers to the number of cells. One to six neurons per animal were recorded. Any electrophysiological measure was obtained by pooling data from at least five animals of each group.

### Drugs

In some cases, animals were intraperitoneally (i.p.) injected with the CB1 receptor antagonist AM251 (6 mg/kg) (AM, Tocris, UK) or with the fatty acid amide hydrolase inhibitor URB597 (0.3 mg/kg) (URB, Alexis, USA). Both drugs were dissolved in a vehicle (VHL), composed by saline with 10% of DMSO and 5% of Tween 80 and administered in an injection volume of 5 ml/kg. Animals used as controls received i.p. the same amount of vehicle. According to their pharmacokinetic properties, drugs were administered 30 min before the behavioral testing [63], [65]. Drugs were administered at dosages reported in literature [26], [39].

### Statistical Analysis

Data presented as mean ± SEM (or ± SD) were tested for normality (Will-Shapiro's test) and homoscedasticity (Levene's test). While frequencies of white choices in S1 and S2 were compared by means of χ^2^ test, the remaining behavioral and electrophysiological data were compared by using analyses of variance (ANOVAs), followed by Tukey's HSD test or paired Student's t-test. Pearson's correlation was run to determine the relationship between white choices in the S2 in A/A Y-Maze and time of contact with the object in OF. The differences were considered significant at the *P*<0.05 level.

## Supporting Information

Materials and Methods S1 **Description of subjects, experimental procedures and global timing of the experimental design are summarized.**
(DOC)

Figure S1 **Flow diagram of the experimental design.** Procedures and global timing are indicated. Out of the 780 adolescent C57BL/6J mice tested in the A/A Y-Maze, 206 animals were used to build the distribution curve of their behavior in response to conflicting stimuli in A/A Y-Maze, while the remaining 574 mice were analyzed for their responses to A/A Y-Maze, Open Field (OF) test and Elevated Plus Maze (EPM). At the end of behavioral testing, Electrophysiological Recordings (ER) were performed from spiny striatal neurons. The behavioral (A/A Y-Maze, OF) and electrophysiological effects of drugs (URB597, URB; AM251, AM) or vehicle (VHL) acting on endocannabinoid system were also analyzed.
(TIF)
