# Supplementary material for: Differences in Spontaneously Avoiding or Approaching Mice Reflect Differences in CB1-Mediated Signaling of Dorsal Striatal Transmission
Source: PLoS One. 2012 Mar 8;7(3):e33260. doi: 10.1371/journal.pone.0033260 (PMC3297636; doi:10.1371/journal.pone.0033260)
Supplement: Materials and Methods S1 — Description of subjects, experimental procedures and global timing of the experimental design are summarized. (DOC) [file pone.0033260.s001.doc]

**Subjects, experimental procedures and global timing**

Seven hundred eighty male adolescent (32 ± 2 pnd) C57BL/6JOlaHsd mice (Harlan, Italy) were used. The animals arrived to our stabulary after weaning (21^th^ pnd). After their arrival from shipment an at least 5-day adjustment period to the new home condition was allowed. The animals were housed four per cage, with food (Mucedola 4RF21, Italy) and water ad libitum, under a 12-h light/dark cycle with light on at 07:00 hours, controlled temperature (22-23°C) and constant humidity (60 ± 5%). All animals of the present research (n = 780) were tested in the A/A Y-Maze. Out of these animals, 206 mice were used to build the distribution curve of the behavior in the A/A Y-Maze. Thus, we found animals belonging to three different categories: - *balancing* (BA) animals whose values corresponding to the mean; - *avoiding* (AV) animals whose values were minus two standard deviations of the mean; - *approaching* (AP) animals whose values were plus two standard deviations of the mean.

Within the remaining 574 animals we found 24 animals (8/category) subsequently tested in the OF test. Two weeks later these animals were sacrificed to make electrophysiological recordings of striatal synapses.

Other 30 mice belonging to AP and AV categories were injected with drugs acting on ECS. Namely, the AV animals treated with URB597 (n=5), AM251 (n=5) or vehicle (n=5) and the AP animals treated with AM251 (n=5), URB597 (n=5) or vehicle (n=5) were re-tested in A/A Y-Maze following the protocol applied in S2. Forty-eighty hours later, the animals were re-injected with the same drugs and tested in the OF test. Two weeks later, the animals were again re-injected with the same drugs and sacrificed to make electrophysiological registrations of striatal synapses.

To better clarify the contribution of anxiety components in the avoidance and approach behaviors other 24 (8/category) AV, BA and AP mice were tested in the S1 of OF and 48 hours later in the EPM test.
