# Supplementary figures and images for: Differences in Spontaneously Avoiding or Approaching Mice Reflect Differences in CB1-Mediated Signaling of Dorsal Striatal Transmission
Source: PLoS One. 2012 Mar 8;7(3):e33260. doi: 10.1371/journal.pone.0033260 (PMC3297636; doi:10.1371/journal.pone.0033260)

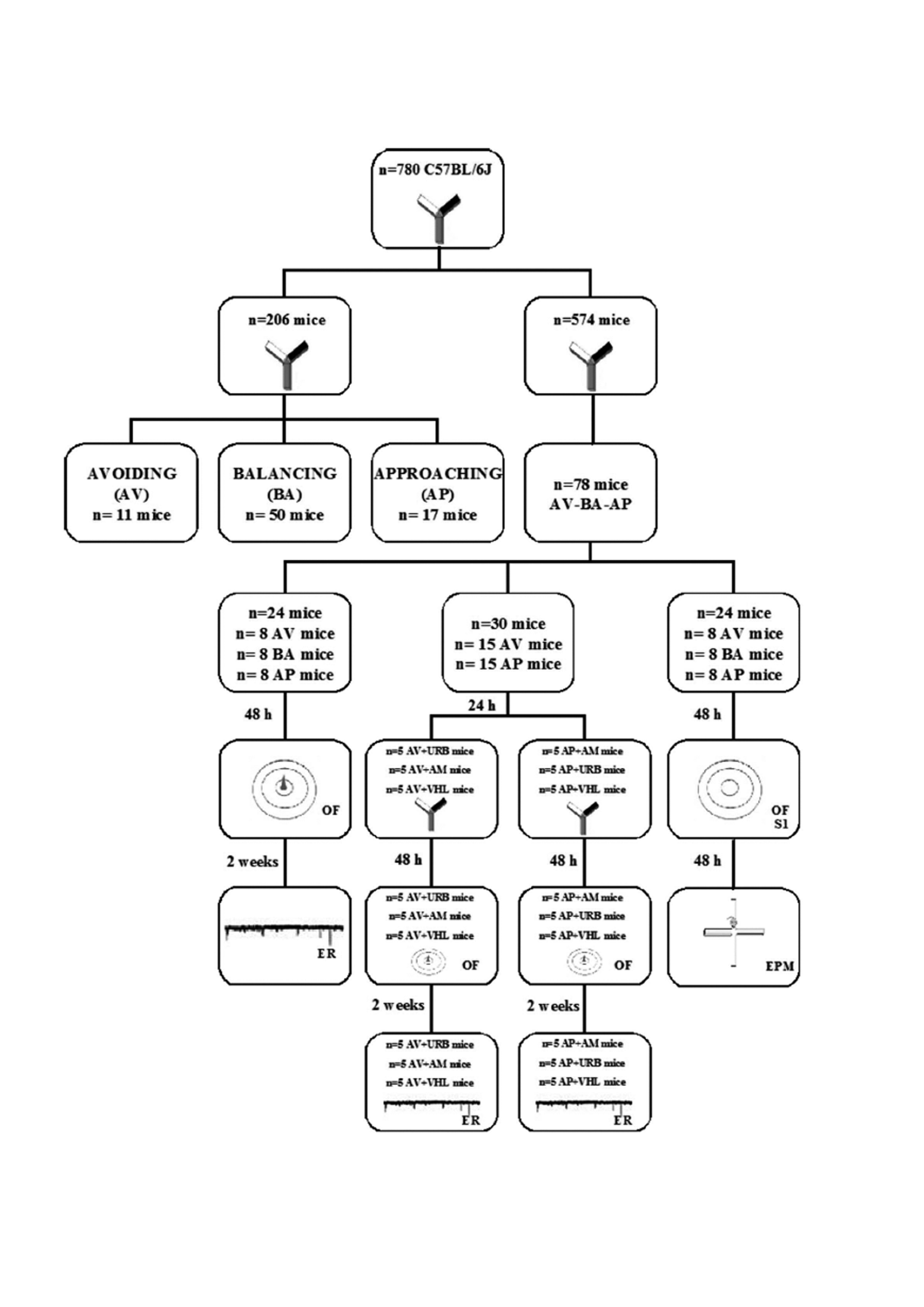

Supplement: Figure S1 — Flow diagram of the experimental design. Procedures and global timing are indicated. Out of the 780 adolescent C57BL/6J mice tested in the A/A Y-Maze, 206 animals were used to build the distribution curve of their behavior in response to conflicting stimuli in A/A Y-Maze, while the remaining 574 mice were analyzed for their responses to A/A Y-Maze, Open Field (OF) test and Elevated Plus Maze (EPM). At the end of behavioral testing, Electrophysiological Recordings (ER) were performed from spiny striatal neurons. The behavioral (A/A Y-Maze, OF) and electrophysiological effects of drugs (URB597, URB; AM251, AM) or vehicle (VHL) acting on endocannabinoid system were also analyzed. (TIF) [file pone.0033260.s002.tif]
